# Aldosterone and angiotensin II induce protein aggregation in renal proximal tubules

**DOI:** 10.1002/phy2.64

**Published:** 2013-09-10

**Authors:** Muhammad U Cheema, Ebbe T Poulsen, Jan J Enghild, Ewout Hoorn, Robert A Fenton, Jeppe Praetorius

**Affiliations:** 1 Department of Biomedicine, Membranes & InterPrET, Health, Aarhus UniversityAarhus, Denmark; 2 Department of Molecular Biology and Genetics, iNano, Science and Technology, Aarhus UniversityAarhus, Denmark; 3 Department of Internal Medicine, Nephrology, Erasmus Medical CenterRotterdam, the Netherlands

**Keywords:** Aggresome, autophagy, protein aggregation, proximal tubules

## Abstract

Renal tubules are highly active transporting epithelia and are at risk of protein aggregation due to high protein turnover and/or oxidative stress. We hypothesized that the risk of aggregation was increased upon hormone stimulation and assessed the state of the intracellular protein degradation systems in the kidney from control rats and rats receiving aldosterone or angiotensin II treatment for 7 days. Control rats formed both aggresomes and autophagosomes specifically in the proximal tubules, indicating a need for these structures even under baseline conditions. Fluorescence sorted aggresomes contained various rat keratins known to be expressed in renal tubules as assessed by protein mass spectrometry. Aldosterone administration increased the abundance of the proximal tubular aggresomal protein keratin 5, the ribosomal protein RPL27, ataxin-3, and the chaperone heat shock protein 70-4 with no apparent change in the aggresome–autophagosome markers. Angiotensin II induced aggregation of RPL27 specifically in proximal tubules, again without apparent change in antiaggregating proteins or the aggresome–autophagosome markers. Albumin endocytosis was unaffected by the hormone administration. Taken together, we find that the renal proximal tubules display aggresome formation and autophagy. Despite an increase in aggregation-prone protein load in these tubules during hormone treatment, renal proximal tubules seem to have sufficient capacity for removing protein aggregates from the cells.

## Introduction

The reabsorption of large amounts of filtered water and solutes by the renal tubule system is regulated by circulating hormones and local mediators (Boron and Boulpaep 2003). However, performing this major task may increase the risk of inappropriately loading the cells with misfolded or damaged proteins. These proteins can form intracellular aggregates, which may prevent the cells maintaining normal cellular functions (Wetzel 1994; Kopito 2003; Yuan et al. 2012a). Aggregation-prone proteins may form due to high protein turnover, which is accompanied by a greater risk of misfolded protein following translation and a higher requirement for “damaged” proteins to be degraded. Furthermore, the required high mitochondrial activity can lead to production of reactive oxygen species (ROS; Yuan et al. 2012b) that could further increase the load of damaged aggregation-prone proteins for the degradation system.

Protein aggregates can form anywhere in the cytoplasm, if the efficiency of the heat shock protein 70 chaperones that normally minimize protein misfolding and aggregation is exceeded (Martin and Hartl 1997). Protein aggregates, to some extent, contain polyubiquitinated proteins marked for proteasomal degradation. However, once proteins are trapped in aggregates the proteasomes are incapable of handling these proteins and an alternative mechanism is activated (Fabunmi et al. 2000). In this alternative mechanism, ataxin-3 cleaves the c-terminal end of the ubiquitin chain, exposing a normally masked domain to the cytoplasm (Mao et al. 2005). HDAC6 then binds to the partly deubiquitinated protein aggregates and links them via dynactin p62 to the dynein motor protein (Hubbert et al. 2002; Kawaguchi et al. 2003; Iwata et al. 2005). Dynein carries the aggregates retrograde toward the microtubule organizing center, where large aggregates called aggresomes are formed (Kopito 2000). The aggresomes, in turn, are cleared by autophagy, that is, they are engulfed by autophagosomes that fuse with lysosomes where lysosomal enzymes effectively degrade the protein load (Mak et al. 2010).

The renal tubules have a high metabolic rate, reflects a high resting O_2_ consumption and the extra energy demand in relation to Na^+^ reabsorption (Boron and Boulpaep 2009). Additionally, the tubules face an even greater stress due to the endocytosis of large amounts of filtered proteins and vitamins – in addition to the high rate of solute and water transport (Boron and Boulpaep 2009). Enhanced autophagy has been reported in disease models with renal damage (Kaushal et al. 2008), but little is known about the capacity of the proximal tubular protein aggresome–autophagosome degradation system under normal conditions and high physiological concentrations of the renal active hormones. Thus, we hypothesized that the hormones aldosterone and angiotensin II would increase the accumulation of aggregation-prone proteins in renal proximal tubules. Classically, angiotensin II acts on AT1 receptors in proximal tubules, where it predominantly affects activity of the ion transport system. Although classical mineralocorticoid receptors (MR) are not expressed in the proximal renal tubules, aldosterone administration increases ROS levels in these tubules (Yuan et al. 2012b). Thus, we employed these hormones as separate means of increasing the generation of aggregation-prone proteins in renal proximal tubules.

## Methods

### Animal experiments

Male wistar rats were administered hormones through subcutaneous osmotic minipumps (Alzet osmotic minipumps, Cupertino, CA). Protocol 1: 50 μg/kg/day aldosterone or vehicle for 7 days with and without 120 mg/kg/day spironolactone (Nielsen et al. 2007). Protocol 2: 200 ng/min/kg angiotensin II or vehicle for 7 days (Kwon et al. 2003). Protocol 3: rats were adrenalectomized, given 12 μg/kg/day dexamethasone as glucocorticoid substitution (van der Lubbe et al. 2011) and then either 50 μg/kg/day aldosterone, or 233 μg/kg/day angiotensin for 8 days. Rats were anesthetized with isofluorane, blood was sampled, and the right kidney removed and processed for immunoblotting. The left kidney was fixed by perfusion with 3–4% paraformaldehyde in 0.01% PBS. The animal experiments were performed according to the license issued by the Danish Ministry of Justice.

### Immunohistochemistry

Fixed kidneys were dehydrated in graded ethanol (70, 96, and 99%) for 2 h each and left overnight in xylene. The tissue was embedded in paraffin wax, cut into 2 μm thick sections on a rotary microtome (Leica Microsystems GmbH, Wetzlar, Germany), and placed on Super Frost slides. Sections were dewaxed in xylene and rehydrated in graded ethanol. Endogenous peroxidase was blocked in 35% H_2_O_2_ in methanol. To retrieve antigens, sections were boiled in a microwave oven in TEG-buffer pH 9 with 10 mmol/L Tris and 0.5 mmol/L EGTA. Aldehydes were quenched in 50 mM NH_4_Cl in PBS, and the sections were blocked in 1% BSA, 0.2% gelatin, 0.05% saponin in PBS. Then, sections were incubated with primary antibody diluted in 0.1% BSA, 0.3% Triton X-100 in PBS overnight at 4°C, and rinsed in 0.1% BSA, 0.2% gelatin, 0.05% saponin in PBS.

For light microscopy, sections were incubated 1 hour with horseradish peroxidase conjugated secondary antibody in 0.1% BSA, 0.3% Triton X 100 in PBS and washed in 0.1% BSA, 0.2% gelatin, 0.05% saponin in PBS before visualization with diaminobenzidine in 35% H_2_O_2_ for 10 min. The sections were counterstained with Mayers hematoxylin and rinsed in running tap water before dehydration in graded ethanol and xylene and mounting with coverslips (Eukitt; CellPath, Newtown, U.K.). For immunofluorescence staining, the blocking of peroxidase was omitted and fluorophore-tagged secondary antibodies were applied. In this case coverslips were mounted with a hydrophilic mounting media containing antifading reagent (Glycergel; Dako, Glostrup, Denmark).

### Antibodies

The primary antibodies utilized are described in Table 1. Secondary antibodies were horseradish peroxidase conjugated goat anti-rabbit IgG or donkey anti-Goat IgG (Dako). For fluorescence detection, donkey anti-rabbit, -goat, or -mouse Alexa Fluor 488, 555, or 633 (Invitrogen, Life Technologies, Carlsbad, CA) were used.

**Table 1:** Antibodies used for immunohistochemistry and/or immunoblotting

Abbreviated name	Target	Company	Host
RPL22/27	H-95	Santa Cruz (sc-21012)	Rb
KRT5	Cytokeratin 5	LSBio (LS-B5496)	Rb
Hsp 70-4	Heat Shock Protein	Epitomics (3143-1)	Rb
Ataxin-3	Polyubiquitin chains	Thermo Scientific (PA5-22369)	Rb
Proteasome 20s	Proteasome marker	Abcam (Ab3325)	Rb
Dynactin p62	Aggresome marker	Santa Cruz (sc-55603)	Mo
HDAC6	Aggresome marker	Santa Cruz (sc-5258)	Gt
LC3	Autophagosome marker	Abcam (Ab58610)	Rb
RPL27	60s Ribosomal Protein L27	ProteinTech (14980-1-AP)	RB
Calbindin-D28K	Calbindin	Fitzgerald (10R-C106a)	Mo
Albumin	Human Serum Albumin	Abcam (ab8940)	Sh
Vimentin	Vimentin	Millipore (MAB3400)	Mo

Rb, rabbit; Mo, mouse; Sh, sheep; Ch, chicken.

### Light microscopy and image processing

Brightfield imaging was performed on a Leica DMRE light microscope with PC APO 63×/1.32–0.6 NA and PC Fluotar 25×/0.75 NA oil immersion objectives, and a Leica DC 300 digital camera. Fluorescence imaging was performed on a Leica DM IRE2 inverted confocal microscope using a Leica TCS SP2 laser module and an HCX PC APO CS 63×/1.32 NA oil objective. Images were acquired with 8-bit image depth, 1024 × 1024 pixel resolution, with an image averaging of six frames. For quantitation of staining intensities, laser power and settings for PMT gain and offset was kept constant for each antibody and adjusted to the brightest section. Image-Pro Analyzer (Media Cybernetics, Silver Spring, MD) and ImageJ (NIH, Bethesda, MD) were used for semi-quantitation and merging the confocal images. For semi-quantitation, the tubular outline was defined, then the cell area was determined, and the background-corrected fluorescence signal determined. The fluorescence signal, particle size, or numbers were then normalized to the total tubule cell area. For calculation of colocalization, the Manders' coefficients were determined using Imaris 5.5 software (Bitplane, Zurich, Switzerland) after thresholding the images. This is the optimal measure of colocalizing spot-like structures from two color channels on a dark background, as all pixels without signal above threshold are ignored (http://www.svi.nl/ColocalizationCoefficients).

### Immunoblotting

The kidneys were homogenized (Ultra-Turrax T8 homogenizer, Fisher Scientific, Waltham, MA) in ice-cold dissection buffer containing 300 mmol/L sucrose, 25 mmol/L imidazole, 1 mmol/L EDTA, 8.5 μmol/L leupeptin, and 1 mmol/L phenylmethylsulfonyl fluoride, pH 7.4. After centrifugation at 4000 g for 15 min at 4°C, the supernatants were spun at 17,000 g for 15 min at 4°C. The 17,000 pellets were dissolved in Tris buffer and the protein concentrations were measured with a BCA protein assay (Pierce, Rockford, IL). The Tris buffer was adjusted to 3% SDS, 8.7% glycerol, bromophenol blue, 30 mg/ml dithiothreitol, and pH 6.8 and heated 15 min at 65°C. Proteins were separated in 12.5% polyacrylamide gels (Criterion gels; Bio-Rad) at 100 V for 70 min and were electrotransferred onto Hybond-ECL nitrocellulose membranes (GE Life Sciences, Amersham, U.K.) for 60 min at 100 V. Membranes were blocked in 5% nonfat dry milk in phosphate buffer consisting of 281 mmol/L Na^+^, 100 mmol/L Cl^−^, 21 mmol/L H_2_PO_4_^−^, 80 mmol/L HPO_4_^2−^, 0.1% Tween 20, and pH 7.5 for 1 h at room temperature and incubated overnight at 4°C with primary antibodies. The antibody–antigen reactions were visualized with enhanced chemiluminescence system (ECL Plus Western Blotting detection system; GE Life sciences) and exposed to photographic film (Hyperfilm ECL; GE Lifesciences). Films were scanned on a flatbed scanner at 8-bit depth and 600 dpi resolution and bands semi-quantified after background subtraction within a linear range using ImageJ software.

### Fluorescence activated particle sorting

Kidney homogenates were labeled by the ProteoStat® Aggresome Detection Kit (Enzo Life Sciences, Farmingdale, NY) after dilution 1:10 in 1X Assay Buffer and processed according to manufacturer's protocol. Labeled particles were sorted using the 488 nm laser on a FACSAria III (BD Biosciences). Particles were first gated according to forward- and side-scatter, then sorted based on fluorescence intensity. Emitted light was collected using a 600/30 nm band pass filter. Sorted particles were collected in 50 mM NH_4_HCO_3_ and stored at -20°C before being concentrated to 25 μl in a speed-vac centrifuge system, and subsequently adjusted to 100 mM DTT, and 50 mM Tris-HCl, pH 8. Samples were boiled for 1 min and allowed to cool on ice for 5 min before added 400 mM freshly made iodoacetamide and incubated 30 min in darkness. Alkylated samples were digested overnight at 37°C by incubating with 25 ng sequence grade trypsin for 16 hours. The resulting peptides were desalted using C_18_ StageTips (Thermo Scientific), and stored at -20°C before Liquid Chromatography Tandem Mass Spectrometry (LC-MS/MS).

### LC-MS/MS analysis and protein identification

Liquid Chromatography Tandem Mass Spectrometry was performed on an EASY-nLC II system (Thermo Scientific) connected to a TripleTOF 5600 mass spectrometer (AB Sciex) equipped with a NanoSpray III source (AB Sciex) and operated under Analyst TF 1.5.1 control. The trypsin digested samples were dissolved in 0.5% formic acid, injected, trapped, and desalted isocratically on a ReproSil-Pur C18-AQ column (5 μm, 2 cm × 100 μm I.D.; Thermo Scientific) after which the peptides were eluted from the trap column and separated on an analytical ReproSil-Pur C18-AQ capillary column (3 μm, 10 cm × 75 μm I.D.; Thermo Scientific) connected in-line to the mass spectrometer at 250 nl/min using a 50 min gradient from 5% to 35% phase B (0.1% formic acid and 90% acetonitrile). An information-dependent acquisition method was employed to automatically run experiments acquiring up to 50 MS/MS spectra per cycle using 2.8 sec cycle times and an exclusion window of 6 sec. The collected MS files were converted to Mascot generic format (MGF) using the AB SCIEX MS Data Converter beta 1.1 (AB SCIEX) and the “proteinpilot MGF” parameters. The peak lists were used to interrogate the Swiss-Prot (version 2012_04, 535,698 sequences) Rattus (7750 sequences) and Homo sapiens (20,250 sequences) databases using Mascot 2.3.02 (Matrix Science). Trypsin was employed as enzyme and allowed one missed cleavage. Carbamidomethyl was chosen as a fixed modification and oxidation of methionine was entered as variable modification. The mass accuracy of the precursor and product ions were 10 ppm and 0.4 Dalton and the instrument setting was specified as ESI-QUAD-TOF. The significance threshold (p) was set at 0.01 and with an ion score cutoff at 30. Mascot results were parsed using the software MS Data Miner (v. 1.0) (Dyrlund et al. 2012), protein hits were automatically validated if they satisfied one of the following criteria: (i) identification based on one or more unique peptides with ion score above or equal to 45 or (ii) identification based on two or more unique peptides with ion score above or equal to 30. For identifications based on only one unique peptide with ion score between 30 and 45, the MS/MS data were manually validated by assignment of significant peaks and occurrence of uninterrupted y- or b-ion series of at least three consecutive amino acids.

### Statistics

Data from semi-quantitative immunoblotting and immunofluorescence histochemistry were tested by two tailed t-tests choosing a significance level of *P* < 0.05.

## Results

### Proximal tubules express the molecular machinery for autophagy of protein aggregates

**Figure 1:**
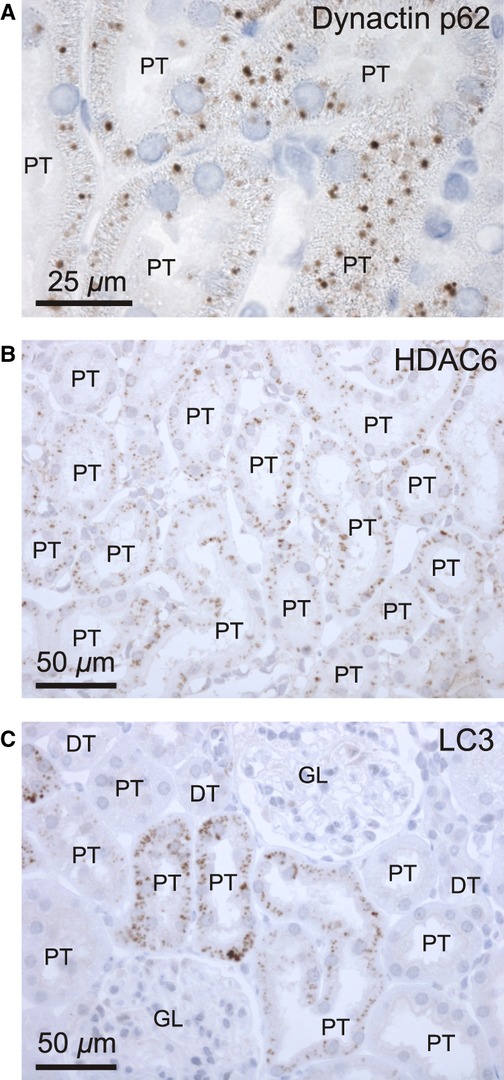
Proximal tubules express the machinery for protein aggregate autophagy. Rat kidney cortex was immunolabeled with markers of various components of the cellular machinery to dispose of protein aggregates by autophagy. (A) Renal cortex immunoperoxidase staining pattern for dynactin p62, which is a linker protein between cargo bound to HDAC6 destined for aggresomes and the motor protein dynein. (B) The renal proximal tubular staining pattern for the aggresome and aggresome cargo marker HDAC6. (C) Autophagosome marker LC3 immunoreactivity in the proximal tubules of renal cortex. Proximal tubules (PT); late distal tubules and connecting tubules (DT), glomeruli (GL).

Distal renal tubules are incapable of producing aggresomes and inducing autophagy when challenged with intracellular protein aggregation (unpublished observations). To assess the proximal tubular capacity for aggresome formation and autophagy, we first stained normal rat tissue for markers of various steps in the process. Dynein motor proteins move in the retrograde direction along the microtubule filaments. Among other types of cargo, dynein-bound dynactin p62 links aggregated protein cargo to the microtubules before transport to the aggresome near the microtubule organizing center. Figure 1A shows that dynactin p62 labeled kidney cortex in punctate pattern. Both large and small dynactin p62 punctae were observed, but the immunolabeling was confined to proximal tubules. The formation of mature aggresomes requires recruitment of HDAC6. As illustrated in Figure 1B, HDAC6 immunostaining was also specific for proximal tubules. For HDAC6, only large punctae were observed consistent with the labeling of mature aggresomes. LC3 is a marker for autophagosomes. Figure 1C shows that autophagosomes develop in proximal tubules of normal rat kidneys, as assessed by the marker protein LC3.

### Aggresomes are formed in normal renal proximal tubules

**Figure 2:**
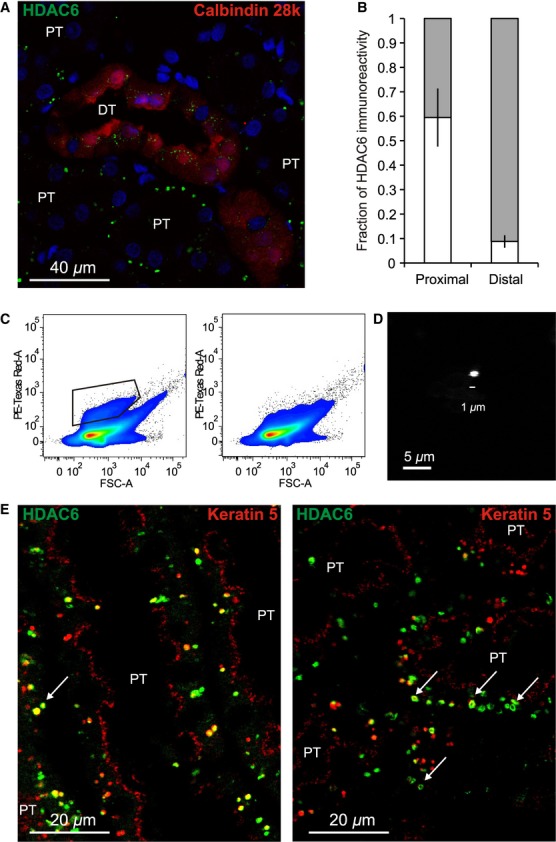
Aggresomes are formed in normal renal proximal tubules. Kidney sections from normal rats were subjected to fluorescence immunohistochemical analysis of HDAC6 distribution. (A) Representative micrograph of renal cortical HDAC6 immunolabeling (green) with colabeling for late distal renal tubules and connecting tubules (Calbindin-D 28k, red) and nuclear counter stain (blue). (B) Quantitation of the distribution of large (>1.5 μm, white boxes) and small (≤1.5 μm, gray boxes) intracellular HDCAC6-positive particles in distal and proximal renal tubules, as indicated. The aggresomes on renal cortex homogenates were fluorescence labeled before fluorescence-activated particle sorting. (C) Scatter diagram showing the fluorescence intensity of aggresome-stained homogenates rat cortex as function of the forward scatter (left panel). The boxed area indicates the fraction of particles chosen for collection. The right panel shows similar profile from unlabeled rat cortex sample. (D) A fraction of the isolate was imaged by confocal microscopy to ascertain the fluorescence level and particle sizes were comparable with labeling in rat kidney. (E) Examples of double immunostaining of rat kidney section with a keratin 5 antibody (red) and HDAC6 (red). Proximal tubule lumen (PT); late distal tubule and connecting tubule lumen (DT), arrows indicate areas with predominantly peripheral staining.

HDAC6 attaches to partly deubiquitinated protein aggregates and transfers these to aggresomes. Figure 2A is a double immunolabeling micrograph of rat cortical HDAC6 labeling and a marker for late distal renal tubules and connecting tubules, Calbindin 28K. HDAC6 labeling of distal renal tubules is confined to small punctae, most likely reflecting binding to protein aggregates but not classical larger aggresomes. Here, the proximal tubules display both small and larger punctae, indicating formation of mature aggresomes. We quantified the sizes of the HDAC6 punctae of proximal and distal tubules in five images from each of five control rats. Figure 2B shows the summarized distribution of large and small punctae in proximal and distal tubules, respectively, with white bars representing the larger punctae. From this it is clear that the proximal tubule has a significantly higher fraction of larger punctae. The number of large HDAC6 punctae in distal tubules is a conservative estimate, as the thresholding of fluorescence images tended to identify groups of bright small punctae as one large HDAC6 positive area.

### Identification and validation of proteins in renal tubular aggresomes

To identify proteins included in the renal proximal tubular aggresomes, fluorescence-labeled aggresomes were subjected to fluorescence-activated particle sorting. Figure 2C demonstrates the entire population of particles from labeled rat cortex (left panel) with the collected population marked with a black line. The right panel shows the population from an unlabeled rat kidney sample. Figure 2D is an inspection of the sorted particles (left panel) by confocal microscopy to ascertain that the fluorescence characteristics and particle sizes are relevant to those of aggresomes in proximal renal tubules (right panel). Table 2 shows the proteins identified by mass spectrometry after aggresome sorting. Keratin type I (10, 17, and 42) and keratin type II (1, 5, 6A, and 75) were among the significant protein identifications. As human and rodent hair and skin keratins and are frequently identified via LC-MS/MS due to contamination, we were cautious of their identification and wanted to independently verify their expression in the tissue. We performed confirmatory immunoblotting and immunohistochemistry for the best hit (keratin 5) in rat kidneys. Figure 2E shows the keratin 5 expression in rat kidney colabeled for HDAC6. The staining illustrates that punctate keratin 5 immunoreactivity is indeed observed in renal proximal tubules similar to HDAC6 and that these proteins partially colocalize in punctate structures. Thus, aggresomes containing keratin 5 are present in renal proximal tubules from control rats. Interestingly, it seems that the keratin 5 and HDAC6 labeling is strongest at the periphery of the punctae.

**Table 2:** Aggresome proteins identified by mass spectrometry

Protein	Score	emPAI
Actin, cytoplasmic 1	529	1.35
Calmodulin	69	0.51
Fructose-bisphosphate aldolase B	197	0.56
Gamma-glutamyltranspeptidase 1	314	0.5
Histone H4	43	1.47
Keratin, type I cytoskeletal 10	813	1.44
Keratin, type I cytoskeletal 17	640	1.27
Keratin, type I cytoskeletal 42	339	0.77
Keratin, type II cytoskeletal 1	697	0.65
Keratin, type II cytoskeletal 5	667	1.86
Keratin, type II cytoskeletal 6A	543	1.07
Keratin, type II cytoskeletal 75	476	0.84
Sodium/potassium-transporting ATPase subunit alpha-1	487	0.61
Superoxide dismutase [Cu-Zn]	82	0.91

### Proteins involved in clearing aggregates only partially colocalize

**Figure 3:**
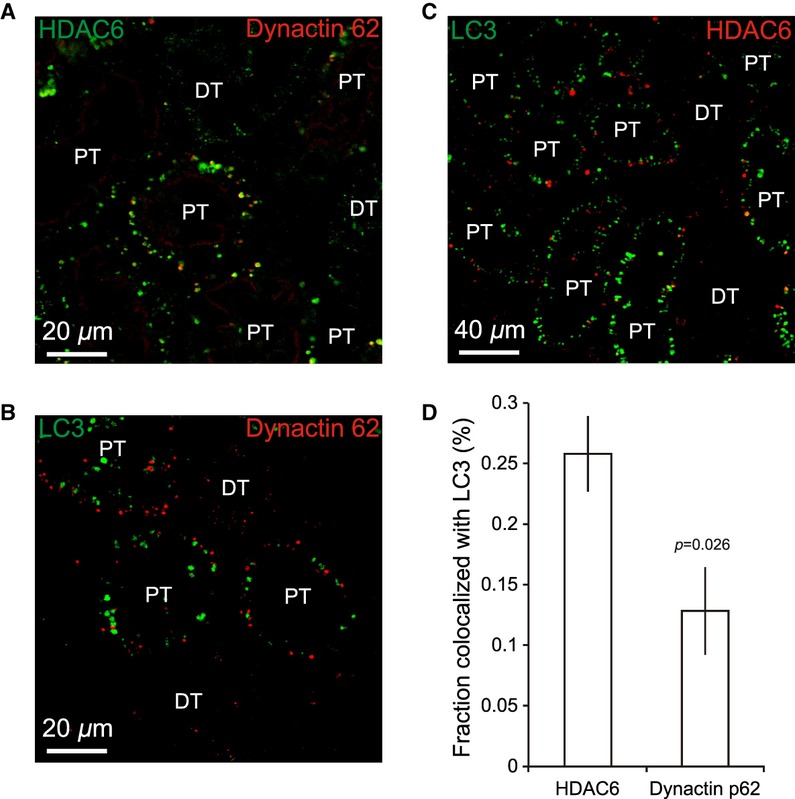
Proteins involved in clearing aggregates only partially colocalize. Double immunolabeling was performed on rat kidney sections to evaluate the relation of the cellular proteins involved in the transfer of protein aggregates from the cytosol via aggresomes to autophagosomes. (A) Representative micrograph of dynactin p62 (red) and HDAC6 (green) colabeling. (B) Immunolabeling for dynactin p62 (red) and LC3 (green). (C) Immunolabeling for HDAC6 (red) and LC3 (green). Proximal tubule lumen (PT); late distal tubule and connecting tubule lumen (DT). (D) Quantitative analysis of the proximal tubular colocalization of LC3 with dynactin p62 and HDAC6, respectively.

Double immunofluorescent labeling was performed to determine whether any of the above markers colocalize in the pathway from protein aggregate via aggresomes to autophagosomes. Extensive colocalization would be expected if the aggresome–autophagy pathway is blocked or compromised. First, we assessed whether dynactin p62 is associated with aggresomes in renal proximal tubules. For this labeling, large HDAC6-positive punctae were used as markers for aggresomes. Figure 3A shows partial colocalization (in yellow) between dynactin p62 and HDAC6 punctae. Thus, the majority of dynactin p62 appear to be en route to the aggresomes or to other cellular structures, while only a minor fraction of dynactin p62 is contained in aggresomes. Dynactin p62 and LC3 double labeling was performed to assess whether a fraction of dynactin p62 punctae were actually included in autophagosomes, which could be HDAC6 negative. Figure 3B shows that the colocalization of dynactin p62 and LC3 is negligible, indicating that the dynactin p62 detaches from the aggresomes along with dynein and microtubules before autophagy of the aggresome. HDAC6 might also either detach from aggresomes or be engulfed into the autophagosome. The double labeling for HDAC6 and LC3 in Figure 3C shows a low degree of colocalization between the two markers, indicating that HDAC6 is cleared along with the aggresome by autophagy. The bar-graph in Figure 3D shows the quantitation of colocalization of LC3 with dynactin p62 and large HDAC6 punctae, as indicated. The autophagosome marker LC3 colocalizes to a higher degree with large HDAC6 punctae (mature aggresomes) than dynactin p62 (cargo transfer protein).

### Aldosterone affects abundance of aggregation-prone proteins in proximal tubules

**Figure 4:**
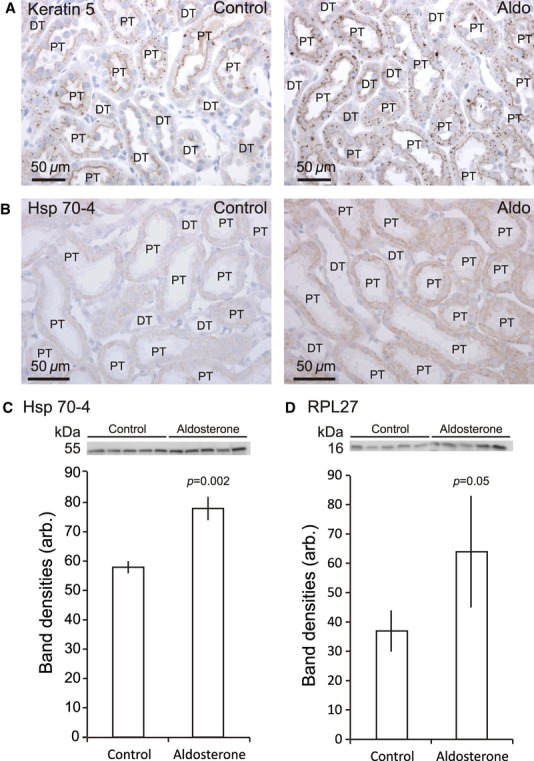
Aldosterone affects abundance of aggregation-prone proteins in proximal tubules. Aldosterone treatment was used as means of promoting renal proximal tubular protein aggregation secondary to reported reactive oxygen species activation. The abundance of potentially aggregated protein and proteins involved in preventing aggregate formation in rat kidneys after 7 days aldosterone administration. (A) Micrographs comparing keratin 5 immunoreactivity in renal cortex from control and aldosterone-treated rats. Proximal tubules (PT), distal tubules and connecting tubules (DT). (B) The expression of the chaperone heat shock protein 70-4 (Hsp70-4) was assessed in kidney sections from control and aldosterone-treated rats. (C) Bar graph showing the densitometry of the full length Hsp70-4 band of 55 kDa from the inserted immunoblot (*P* < 0.05, *n* = 5). (D) Immunoblot and semi-quantitation of RPL27 protein expression in whole kidney from control and aldosterone-treated rats.

Overexpression of proteins, protein damage, or compromised degradation leads to formation of inclusion bodies due to a propensity for improper protein folding. Aldosterone is reported to increase ROS production in proximal tubules even though these tubules do not express the MR and may serve as a model for increased protein damage. Thus, we initially assessed whether aldosterone induced increased keratin 5 abundance in protein aggregates by immunohistochemistry, as preliminary quantitative mass spectrometry of FACS sorted aggresomes indicated that aldosterone induced an increase in renal aggresomal keratin 5 expression. Figure 4A shows that the punctate keratin 5 immunoreactivity observed in controls (left panel) was enhanced by aldosterone treatment (right panel). The heat shock protein 70 (hsp 70) family of chaperones sequester misfolded or damaged protein, thereby preventing them from aggregation. Hsp 70-4 protein labeling was faint or absent in proximal tubule of control rats (Figure 4B, left panel), whereas prominent hsp 70-4 immunoreactivity was observed in kidney sections from aldosterone-treated rats (Figure 4B, right panel). Hsp 70-4 abundance was also analyzed by immunoblotting, in order to verify the apparent difference in expression among the two groups. The immunoblot showed a hsp 70-4 band of approximately 55 kDa (Figure 4C, blot and bar graph), and semi-quantitation showed an approximately 30% increase in hsp 70-4 abundance by aldosterone compared with controls (*n* = 5, *P* < 0.05). Our previous study revealed an aldosterone-induced accumulation of the ribosomal protein RPL22 in distal tubules. An antibody reacting with both RPL22 in distal tubules and the corresponding RPL27 in proximal tubules and a separate RPL27-specific antibody did not convincingly stain proximal tubules of control or aldosterone-treated rats (not shown). However, immunoblot analysis with the RPL27-specific antibody demonstrated that aldosterone induced an approximately 60% increase in RPL27 protein abundance in rat kidney cortex, as shown in Figure 4D.

### Aldosterone induces punctate ataxin-3 labeling in proximal tubules

**Figure 5:**
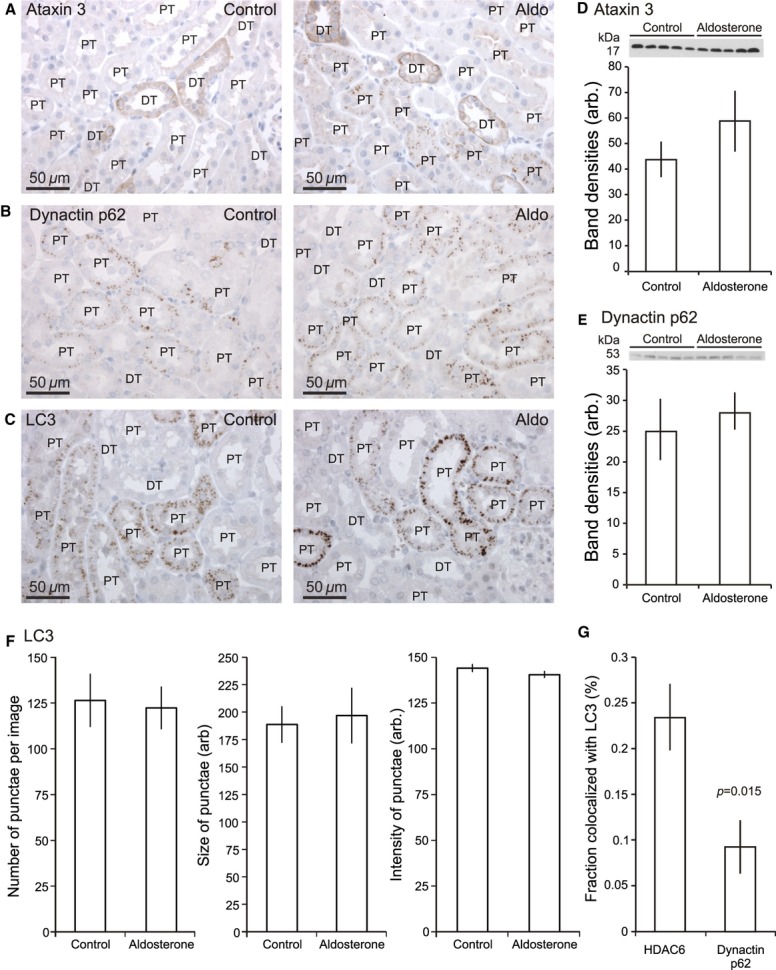
Effect of aldosterone on expression of proteins involved in aggregate handling. For transfer of aggregated proteins to aggresomes, a partial deubiquitination by ataxin-3 must first occur. (A) Ataxin-3 immunoreactivity in control and aldosterone (Aldo)-treated rat kidney sections. Proximal tubules (PT); distal tubules and connecting tubules (DT). (B) Micrographs comparing dynactin p62 labeling in renal cortex from control and aldosterone-treated rats. (C) LC3 immunoreactivity in proximal tubules in control and aldosterone-treated rat kidney sections. (D) Bar graph showing the densitometry of the 17 kDa ataxin-3 band from the inserted immunoblot (*P* > 0.05, *n* = 5). (E) Bar graph shows the semi-quantitation of immunoblot analysis of the 53 kDa dynactin p62 band (insert). (F) The bar graphs show the quantitation of the number, the mean area, and the fluorescence intensity of the renal cortical LC3 staining in the two experimental groups. (G) Quantitative analysis of the proximal tubular colocalization of LC3 with dynactin p62 and HDAC6, respectively, in aldosterone-treated rats.

Ataxin-3 is a deubiquitinase which only partially cleaves the ubiquitin chain from proteins and exposes the c-terminus of ubiquitin to the cytosol instead of the usual *n*-terminal. In control kidney sections, ataxin-3 immunoreactivity was observed selectively in the basolateral domain of the distal renal tubules (Figure 5A, left panel). By contrast, punctate immunolabeling was observed in the proximal tubules of kidneys from aldosterone-treated rats, indicating accumulation of the enzyme in aggregates destined for the aggresomes (Figure 5A, right panel). Kidney homogenates from control and aldosterone-treated rats were also immunoblotted for ataxin-3 abundance and quantified. There was no significant change in the total renal abundance of the 17-kDa band representing ataxin-3 (Figure 5D, *n* = 5, *P* > 0.05). Figure 5B illustrates the renal cortical dynactin p62 staining in control and aldosterone-treated rats. Aldosterone did not alter the dynactin p62 staining pattern or intensity, a result that was confirmed by immunoblotting (Figure 5E, *n* = 5, n.s.). Similar results were obtained for HDAC6 (not shown). Immunostaining for the autophagy marker LC3 (Figure 5C) was similar between the groups. Confirmatory immunoblot analysis was not possible with the anti-LC3 antibody, therefore semi-quantification was performed on fluorescence-labeled kidney sections. The bar graphs of Figure 5F shows that neither the size and number nor the staining intensity of autophagosomes was affected by aldosterone administration (*n* = 5, n.s.). The bar-graph in Figure 5F shows that in aldosterone-treated rat kidneys LC3 colocalizes less with dynactin p62 than with HDAC6, as described for the control rat kidneys in Figure 3D.

### Angiotensin II induces protein aggregation in renal proximal tubules

**Figure 6:**
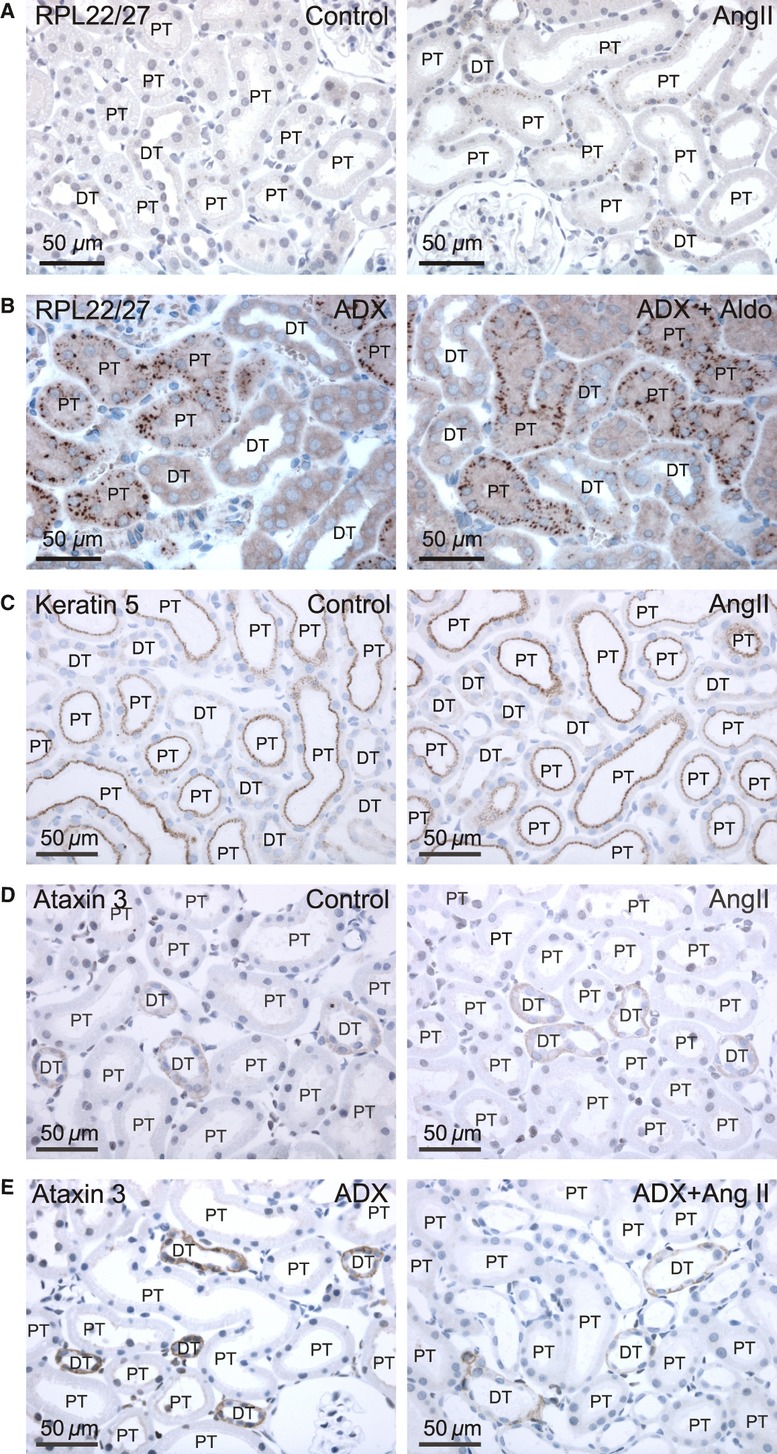
Protein aggregation in renal proximal tubules after angiotensin II treatment. Angiotensin was administered to rats to increase cellular protein turnover in proximal tubules, and aggregation-prone protein content was analyzed by immunohistochemistry. Angiotensin II was elevated by either infusion from subcutaneous minipumps or by adrenalectomy with glucocorticoid substitution. (A) Representative example of the RPL22/27 immunostaining in the kidney cortex from a control and angiotensin II (AngII)-treated rats, as indicated. PT denotes proximal tubules, while DT marks distal tubules and connecting tubules. (B) Similar RPL22/27 staining in sections from dexamethasone-treated adrenalectomized rats (ADX) and from similar rats with aldosterone and dexamethasone coadministration (ADX+Aldo). (C) Micrographs comparing keratin 5 immunoreactivity in renal cortex from control and angiotensin II-treated rats. (D) Immunolabeling for ataxin-3 in the angiotensin II versus vehicle administration model, as indicated. (E) Similar staining in model of dexamethasone-corrected adrenalectomy with and without angiotensin II administration (ADX+AngII), as indicated.

Angiotensin II increases the transport activity of proximal tubules and was employed as another way to increase cell stress. To rule out an effect of angiotensin II on plasma aldosterone levels, angiotensin II was administered alone, or to rats that had undergone adrenalectomy (ADX). Rat kidney sections were labeled with the various antibodies assessed above. In control animals RPL22/27 immunoreactivity was absent from both proximal tubules and distal tubules (Figure 6A, left panel). Angiotensin II-treated rats, with reported high circulating levels of angiotensin II and aldosterone (Kwon et al. 2003; Nielsen et al. 2007), displayed a punctate intracellular staining pattern in both proximal and distal renal tubules (Figure 6A, right panel). In contrast, in kidneys from ADX rats, punctuate RPL22/27 immunolabeling was observed only in proximal tubules (Figure 6B, left panel). Infusion of aldosterone to similar ADX rats (Figure 6B, right panel) reintroduced the pattern of punctate immunoreactivity in distal renal tubules as observed in Figure 6A (left panel). Thus, it appears that distal renal accumulation of RPL22/27 is specific to elevated aldosterone levels, whereas proximal tubular RPL22/27 accumulation is only induced by angiotensin II. We immunolabeled kidney sections from control and treated rats for this protein to assess whether angiotensin II also affected keratin 5 aggregation in proximal tubules. As judged from the micrographs in Figure 6C, angiotensin II did not markedly change keratin 5 distribution or staining intensity (*n* = 5). None of the animal models with altered angiotensin II levels induced ataxin-3 punctae in the proximal tubules (Figure 6D and E), suggesting that aldosterone is required for induction of ataxin-3 in the proximal tubule. Immunostaining indicated no changes in Dynactin p62, HDAC6, and LC3 staining patterns and intensities by angiotensin II treatment (not shown).

### Albumin content in renal proximal tubules unaltered after hormone administration

**Figure 7:**
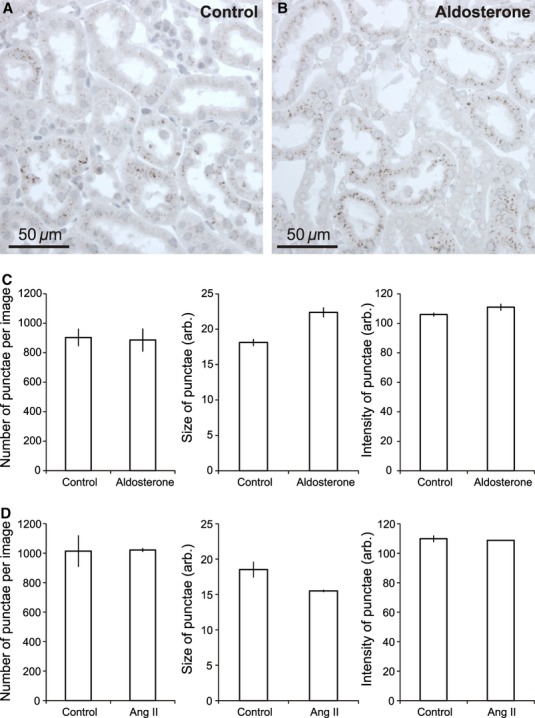
Albumin content in renal proximal tubules unaltered after hormone administration. Glomerular albumin filtration has been reported for prolonged elevation of aldosterone and angiotensin II. Albumin hyperfiltration induces the endocytotic reabsorption of the protein in proximal tubules. Renal cortex was immunostained for albumin to assess whether this mechanism was altered in our models and potentially challenge the lysosomal system and thereby eventually compromise autophagy. (A) Micrograph of renal cortical albumin staining pattern in control rats. (B) Similar staining in the renal cortex of an aldosterone-treated rat. (C) Quantitation of the number, mean area, and fluorescence intensity in control and aldosterone-treated rats, respectively (*n* = 5). (D) Similar quantitation in control and angiotensin II-treated rats.

Elevated plasma levels of both aldosterone and angiotensin II are known to enhance protein filtration in the renal corpuscules. Up to a certain limit, all filtered albumin is endocytosed by the proximal tubular cells. Increased endocytosis leads to augmented degradation of filtered and endocytosed protein by the lysosomes in the proximal tubules. This could potentially decrease the capacity for autophagy, which depends on functional lysosomes. We verified albumin endocytosis in proximal tubules by immunolabeling kidney sections from aldosterone or angiotensin II-treated rats. Compared to controls, immunoreactive albumin was not increased in renal proximal tubule cells by aldosterone treatment (Figure 7A). Analysis of fluorescence-stained rat kidney sections showed that neither the number, the size, nor the staining intensity of albumin were changed by elevated aldosterone (Figure 7C) or angiotensin II (Figure 7D). Furthermore, protein levels in urine were unaffected by aldosterone administration (*n* = 5, *P* = 0.94). Neither protocol was associated with expression of the mesenchymal marker vimentin, as we previously observed for aldosterone-treated rats in distal tubules (not shown).

## Discussion

In this study, we assessed the ability of the renal proximal tubular machinery to dispose of aggregated proteins under normal conditions and after affecting cell function by administration of the hormones aldosterone and angiotensin II. The main finding was that proximal tubules in normal rats form aggresomes and present autophagosomes in the cytoplasm. Furthermore, the proximal tubules do not increase their capacity for aggresome formation or autophagy following increased protein synthesis via aldosterone and angiotensin II.

Both renal proximal and distal tubules have a large content of mitochondria and a high metabolic rate to sustain the active transport of solutes across the epithelia (Boron and Boulpaep 2009). The distal tubules and connecting tubules are incapable of forcing aggresomes and autophagosomes despite increased protein aggregation (authors unpublished observations). This aggregation developed alongside a focal epithelial to mesenchymal transition, as judged by the induction of tubular vimentin expression. Compared to the distal tubule, we here report that proximal tubules harbor the relevant molecular machinery for aggresome formation and an intrinsic capacity of autophagy. We speculate whether this is a reflection of a constant cell stress on the renal proximal tubules. The cells of these tubules simultaneously reabsorb the majority of filtered water and simple solutes, and endocytose the macromolecules, such a proteins and vitamins, by receptor-mediated endocytosis (Boron and Boulpaep 2009).

Using LC-MS/MS we determined that the proximal tubular cell aggresomes contain key proteins previously identified in aggresomes formed in other cell types, such as specific keratins (Riley et al. 2002; Rogel et al. 2010). These keratins might help building a filamentous cage around aggresome or aggregates, thereby antagonizing the potential cell toxic effect of protein aggregation (Johnston et al. 1988; Garcia-Mata et al. 2002; Taylor et al. 2003). In agreement with such function, we actually find both keratin 5 and HDAC6 on the outside of aggresomes in some micrographs. Interestingly, superoxide dismutase was also identified in the aggresomes. The enzyme is an important antioxidant, protecting cellular molecules from superoxide, one of the major ROS. Fructose-bisphosphate aldolase B is involved both in glycolysis and gluconeogenesis (Baynes and Dominiczak 1999) and is likely to be a highly abundant protein in the proximal tubules. Other proteins identified, include the abundant proteins actin (cytoskeletal) and Na,K-ATPase alpha subunit (plasma membrane protein), of which the latter just as fructose-bisphosphate aldolase may be induced to accommodate the higher functional level of proximal tubules by hormonal treatment.

In our colocalization assay for the aggregate–aggresome–autophagosome axis, dynactin p62 is largely excluded from autophagosomes. However, the protein partly colocalize with HDAC6 suggesting that one pool of dynactin p62 is *en route* to or reside in mature aggresomes and the remainder is occupied by transport of other intracellular cargo. The interpretation is consistent with dynactin p62 being involved in many cellular trafficking processes of which transport to aggresomes is only one example (Garces et al. 1999; Corboy et al. 2005; Lee et al. 2010). HDAC6 is not to the same extent excluded from the autophagosomes, and the partial colocalization of the large HDAC6 punctae with LC3 indicates the existence of aggresomes not yet engulfed by autophagosomes.

Elevated levels of aldosterone or angiotensin II increased the abundance of certain proteins, which are indicative of an elevated risk of protein aggregation. For aldosterone, ribosomal RPL27 and keratin 5, and Hsp 70-4 were increased in abundance. Furthermore, the deubiquitinase ataxin-3 appeared in a more punctate pattern in the proximal tubules after aldosterone administration. Angiotensin II seemed to have a more modest effect on protein aggregation and only RPL27 accumulated in intracellular punctae. Nevertheless, the protein accumulation did not lead to changes in the machinery of aggresome formation or autophagy, as observed elsewhere (Johnston et al. 1998). This indicates that the capacity for this type of protein degradation is sufficiently high in proximal tubules and is not compromised by any increased intracellular load of aggregation-prone proteins. Consistent with unaffected tubular functionality, we did not observe any signs of increased apoptosis or epithelial to mesenchymal transition by the elevated hormone levels. The importance of efficient renal protein degradation is stressed by the observation that autophagy protects proximal tubular cells from apoptosis in a model of cisplatin induced nephrotoxicity (Kaushal et al. 2008).

The question remains how aldosterone inflicts changes in proximal renal tubular function? These tubules do not express the MR for classical genomic mineralocorticoid action (Ackermann et al. 2010). However, recent reports demonstrate an induction of ROS production in proximal tubules by elevated aldosterone levels (Yuan et al. 2012a). The effect could arise from either a defective renal filtration barrier as observed by long-term elevated aldosterone and albumin hyperfiltration (Sangalli et al. 2011; Nielsen et al. 2012) or nongenomic effects of aldosterone possibly mediated by the proposed membrane-bound estrogen and aldosterone receptor GPER1/GPR30 (Gros et al. 2011). The hyperfiltration hypothesis is not supported by elevations in intracellular albumin contents or urinary albumin levels in our experiments. Furthermore, GPER seems to be expressed mainly in the distal part of the renal tubular system and is thus unlikely to serve as an alternative MR in proximal tubules (Grimont et al. 2009). As the aldosterone-mediated effects are blocked by spironolactone, an alternative speculation is that aldosterone affects proximal tubules secondary to its effect on the distal tubules and connecting ducts. By contrast, Angiotensin II is known to act on proximal tubules via AT1 receptors and increase ion transport activity through activation of NHE3, NBCe1, and Na,K-ATPase (McDonough 2010). In addition, the expression level of proximal tubular NHE3 is increased by angiotensin II, whereas the Na,K-ATPase expression remains unchanged (Kwon et al. 2003).

In conclusion, we find evidence that renal proximal tubules, in contrast to the distal tubules, contain the molecular machinery for degrading intracellular protein aggregates. Under basal conditions, the proximal tubules contain aggresomes and autophagosomes. The proximal tubules do not respond with increased formation of aggresomes or autophagy although both aldosterone and angiotensin II increase the abundance of proteins indicating an increased risk of aggregate formation. These tubular cells seem to contain machinery sufficient for dealing with hormone-induced protein load for degradation.
