# Corrigendum

**DOI:** 10.1002/phy2.251

**Published:** 2014-02-17

## Introduction

Aldosterone and angiotensin II induce protein aggregation in renal proximal tubules

Muhammad U. Cheema, Ebbe T. Poulsen, Jan J. Enghild, Ewout Hoorn, Robert A. Fenton & Jeppe Praetorius

*Physiol Rep*, 1 (4), 2013, e00064, doi: 10.1002/phy2.64

The author's name, Ewout Hoorn, has been changed to Ewout J. Hoorn.

